# Roles of the Kinase TAK1 in TRAF6-Dependent Signaling by CD40 and Its Oncogenic Viral Mimic, LMP1

**DOI:** 10.1371/journal.pone.0042478

**Published:** 2012-07-30

**Authors:** Kelly M. Arcipowski, Gail A. Bishop

**Affiliations:** 1 Interdisciplinary Graduate Program in Molecular and Cellular Biology, University of Iowa, Iowa City, Iowa, United States of America; 2 Interdisciplinary Graduate Program in Molecular and Cellular Biology, Departments of Microbiology and Internal Medicine, University of Iowa, Iowa City, Iowa, United States of America; 3 Veterans Affairs Medical Center, Iowa City, Iowa, United States of America; University of Amsterdam Academic Medical Center, Netherlands

## Abstract

The Epstein-Barr virus (EBV)-encoded protein latent membrane protein 1 (LMP1) is essential for EBV-mediated B cell transformation and plays a critical role in the development of post-transplant B cell lymphomas. LMP1 also contributes to the exacerbation of autoimmune diseases such as systemic lupus erythematosus (SLE). LMP1 is a functional mimic of the tumor necrosis factor receptor (TNFR) superfamily member CD40, and relies on TNFR-associated factor (TRAF) adaptor proteins to mediate signaling. However, LMP1 activation signals to the B cell are amplified and sustained compared to CD40 signals. We previously demonstrated that LMP1 and CD40 use TRAF molecules differently. Although associating with CD40 and LMP1 via separate mechanisms, TRAF6 plays a significant role in signal transduction by both. It is unknown whether TRAF6 mediates CD40 versus LMP1 functions via distinct or shared pathways. In this study, we tested the hypothesis that TRAF6 uses the kinase TAK1 to trigger important signaling pathways following both CD40 and LMP1 stimulation. We determined that TAK1 was required for JNK activation and interleukin-6 (IL-6) production mediated by CD40 and LMP1, in both mouse and human B cells. Additionally, TRAF3 negatively regulated TRAF6-dependent, CD40-mediated TAK1 activation by limiting TRAF6 recruitment. This mode of regulation was not observed for LMP1 and may contribute to the dysregulation of LMP1 compared to CD40 signals.

## Introduction

Epstein-Barr virus (EBV), the causative agent of infectious mononucleosis [1], [2], latently infects >90% of humans [3]. EBV reactivation in immunocompromised individuals is strongly associated with various malignancies, most notably Burkitt's lymphoma, Hodgkin's disease, nasopharyngeal carcinoma, and post-transplant lymphomas/lymphoproliferative disease (PTLD) [2]–[7]. Recent reports also suggest a role for EBV in diffuse large B cell lymphomas [8]. Additionally, transient EBV reactivation is observed in autoimmune diseases, especially systemic lupus erythematosus (SLE) and rheumatoid arthritis (RA) [9], [10]. EBV preferentially establishes latency in memory B cells, but LMP1 is not expressed in infected cells unless EBV partially emerges from latency, usually in the context of immunosuppression or autoimmunity [1], [7], [11]. LMP1 is expressed in most EBV-associated malignancies and PTLD, and is required for EBV-mediated B cell transformation [12]. LMP1 has also been implicated in the exacerbation of SLE [13], [14].

LMP1 is a 386-amino acid integral membrane protein consisting of a short cytoplasmic (CY) N-terminal domain, six transmembrane (TM) domains, and a long CY C-terminal domain [15], [16]. The N-terminus anchors LMP1 to the plasma membrane and controls LMP1 processing [15]–[17]. The TM domains spontaneously self-aggregate and oligomerize within the plasma membrane, promoting ligand-independent, constitutive activation of signals terminated by the continuous and rapid processing of LMP1 [16]–[18]. Two sub-domains within the C-terminus, C-terminal activating region (CTAR) 1 and CTAR2, are required for LMP1 signaling [15], [17], [19]. Our lab demonstrated in B cell lines and primary B cells that the C-terminal domain is both required and sufficient to mediate most LMP1 functions [2], [20]. In particular, hybrid receptors containing the LMP1 C-terminal domain linked to other external domains mimic LMP1 signaling, including early pathway activation and downstream B cell effector functions [2], [16], [18], [20]–[23].

LMP1 functionally mimics the tumor necrosis factor receptor (TNFR) superfamily member CD40, an activating receptor constitutively expressed on B cells, dendritic cells, and macrophages [24]. LMP1 and CD40 signaling lead to the early activation of kinases and NF-B, followed by downstream events including upregulation of costimulatory and adhesion molecules, and production of pro-inflammatory cytokines [24]. However, LMP1 signals, both early kinase activation and downstream B cell effector functions, are amplified and sustained compared to CD40 [20], [21], [24], [25]. LMP1 and CD40 lack enzymatic activity [24] and use TNFR-associated factor (TRAF) adaptor proteins to mediate signaling, but utilize TRAFs 1, 2, 3, 5, and 6 in distinct and sometimes contrasting ways [12], [18]. TRAF1 and TRAF2 cooperate to promote CD40-mediated JNK and NF-κB activation, but these same LMP1-mediated pathways do not require TRAFs 1 or 2 [26]. TRAF3 negatively regulates CD40 signaling to B cells, but serves as an important positive mediator of LMP1 signaling [15], [18]. TRAF5 modestly enhances CD40-mediated surface molecule upregulation and IgM production, but TRAF5 deficiency has no detectable effect on the activation of CD40-mediated early signaling pathways [27], [28]. In contrast, TRAF5 is required for LMP1-mediated JNK and Akt activation in B cells, as well as IL-6 production [22]. TRAF6 plays an important role in many CD40 functions, binding to CD40 at a membrane-proximal site distinct from the distal site shared by the other TRAFs [24], [29]. TRAF6 is also essential for LMP1-mediated B cell activation [12]. However, it associates with LMP1 via the shared TRAF1/2/3/5 binding site [12], and thus may interact with other TRAFs in a manner distinct from that used in CD40 signaling..

We previously showed that TRAF6 is required for activation of transforming growth factor-β (TGF-β)-activated kinase 1 (TAK1) following LMP1 stimulation [12]. TAK1 is a mitogen-activated protein kinase kinase kinase (MAP3K) important in proinflammatory pathways, particularly those mediated by JNK and NF-κB [30]–[33]. TAK1 is utilized by a variety of receptors, including TGF-βR, IL-1R, and Toll-like receptors. Signaling through these receptors induces TAK1 autophosphorylation and subsequent activation [30], [32], [34]. Due to its role in JNK and NF-κB activation, TAK1 is a potential therapeutic target in a variety of cancers, as these survival pathways are often dysregulated in tumor cells [35]–[39].

TAK1 deficiency impairs B cell proliferation in response to CD40 stimulation [40], but its impact on upstream signaling pathways is unknown. Disagreement between different studies leaves unresolved the question of whether TAK1 is important for LMP1-mediated B cell activation [19], [41]. Prior studies investigating TAK1 in LMP1 signaling were conducted in a transformed adenocarcinoma cell line or mouse embryonic fibroblasts, with exogenously overexpressed proteins [19], [41]. In overexpression systems, non-physiological levels of signaling proteins can produce abnormal pathway activation, confounding data interpretation. We have also demonstrated that TRAF functions can differ depending upon cell type and receptor [42]. Thus, we used a primary target of EBV effects *in vivo*, B cells, and studied proteins expressed at physiological levels.

Given the requirement for TRAF6 in LMP1 functions, including TAK1 activation, and the critical role for TRAF6 in CD40 functions [12], [29], [43], [44], we hypothesized that TRAF6 uses TAK1 to trigger some or all of its signaling pathways following CD40 or LMP1 activation. We examined primary B cells from wild type (WT) or mCD40LMP1 transgenic (Tg) mice, and a complementary model of mouse B cell lines stably expressing a hybrid receptor consisting of the extracellular (EC) and TM domains of mouse (m) or human (h) CD40 attached to the CY domain of LMP1. The hCD40LMP1 molecule allows deliberate initiation of early LMP1 signaling, separate from endogenous mCD40, in stably transfected mouse B cell lines [16]. WT LMP1 signals in a constitutive manner, complicating analysis of early signaling events [12], so the ability to control initiation of LMP1 signals is very important. This CD40LMP1 chimera accurately models WT LMP1 signals in mouse and human B cells, and in mice *in vivo* [16], [23], [45]. To inhibit TAK1 function, we used the well-characterized and widely-used TAK1-specific inhibitor 5Z-7-Oxozeaenol [31], which prevents TAK1 autophosphorylation and activation, and cell lines stably expressing inducible kinase-dead TAK1 (dominant-negative TAK1, DN-TAK1) [2], [29], [30], [46], [47]. To address the roles of specific TRAF molecules in TAK1 activation, we employed mouse B cell lines sufficient or deficient in TRAF3 or TRAF6 [18], [26], [29]. Results presented here indicate that TAK1 was required for a subset of both CD40- and LMP1-mediated signaling pathways, and the downstream function of IL-6 production. Our findings also reveal a novel CD40-specific inter-TRAF interaction that impacts TRAF6-dependent TAK1 activation.

## Results

### IL-6 production

**Figure 1:**
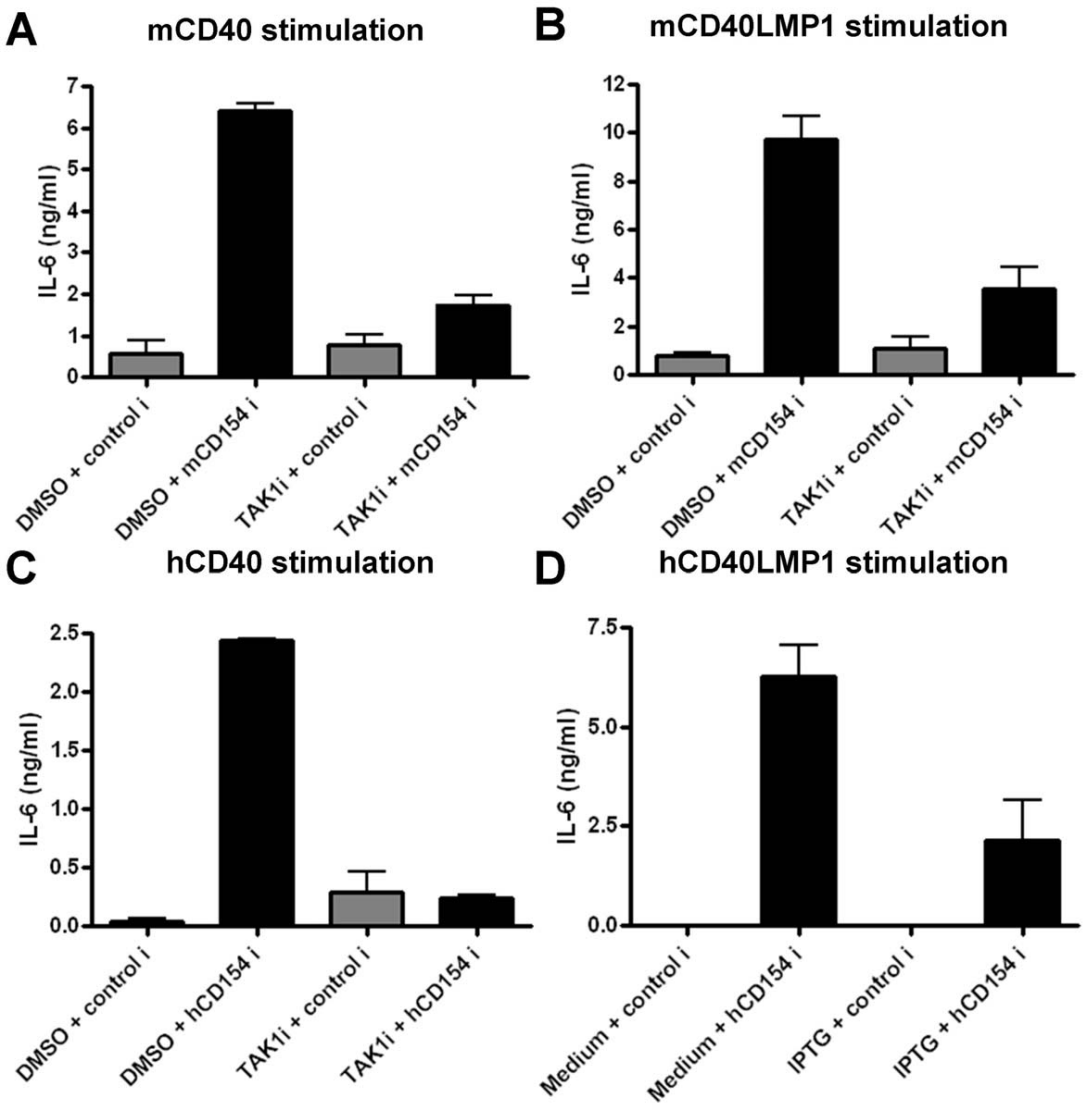
Role of TAK1 in IL-6 production by CD40 and LMP1. A) WT mouse splenic B cells were treated with DMSO or TAK1i prior to stimulation with control insect cells (control i) or those expressing mCD154 (mCD154 i), as described in Methods. Cells were cultured for 24 h and supernatants were collected for IL-6 ELISAs. B) Similar to panel *A*, except that B cells were from a mCD40LMP1 Tg mouse. C) Similar to panel *A*, except that human B cells were stimulated. D) Similar to panel *A*, except that CH12.hCD40LMP1 mouse B cells were treated with medium alone (control) or IPTG to induce DN-TAK1 prior to stimulation. Data in all panels are mean values ± SEM of triplicate samples, and are representative of at least two independent experiments.

IL-6 is an important proinflammatory cytokine produced by B cells in response to signals by CD40 and LMP1. B cells require a membrane-bound form of the CD40 ligand CD154 to induce IL-6 production [24], [48]. Inhibiting TAK1 activation abolished the ability of both endogenous CD40 and CD40LMP1 to mediate IL-6 production in mouse B cells (Fig. 1A & B), as well as human B cells (Fig. 1C). The differences in IL-6 production were not due to increased cell apoptosis induced by TAK1i, as assessed by PI staining and flow cytometry (data not shown). As a complementary approach, we examined mouse B cells expressing a kinase-dead DN-TAK1 [30], [46], [47] via an IPTG-inducible expression system [2], [29]. Corroborating results with TAK1i, DN-TAK1 expression abrogated hCD40LMP1-induced IL-6 (Fig. 1D). Thus, TAK1 was required for CD40- and LMP1-mediated IL-6 production by B cells.

### Antibody production

**Figure 2:**
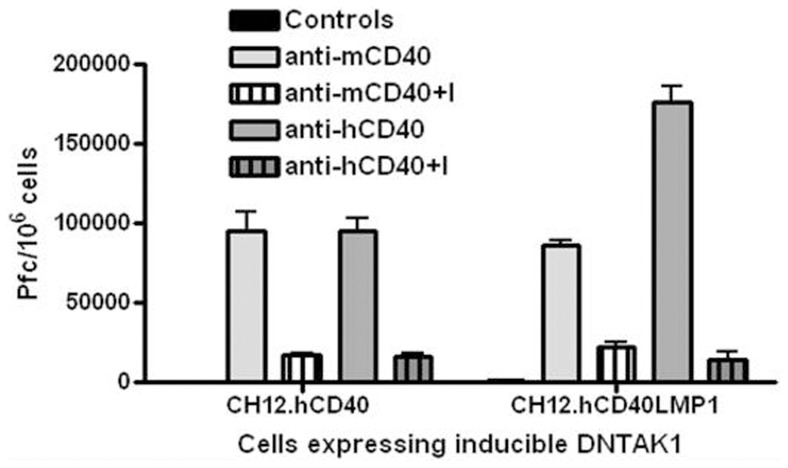
Requirement of TAK1 in CD40- and LMP1-mediated Ab production. CH12.hCD40 or CH12.hCD40LMP1 cells were treated with medium alone or IPTG for 18 h to induce DN-TAK1 prior to stimulation with anti-CD40 Abs, where anti-mCD40 Ab induced signaling by endogenous mCD40, and anti-hCD40 Ab induced signaling via either hCD40 (left) or hCD40LMP1 (right). After 72 h, samples were assayed for IgM production by hemolytic plaque assay. Data are presented as plaque-forming cells (Pfc, IgM-producing cells) per 10^6^ viable recovered cells, mean values ± SEM of replicate samples, and are representative of 3 independent experiments.

Both CD40 and LMP1 induce antibody production by B cells [2]. To examine the requirement of TAK1 in this important function of B cell differentiation, mouse B cells inducibly expressing DN-TAK1 were stimulated through endogenous mCD40, hCD40, or hCD40LMP1 (Fig. 2). DN-TAK1 expression significantly reduced IgM production stimulated by both CD40 and LMP1.

### Activation of early signaling pathways

**Figure 3:**
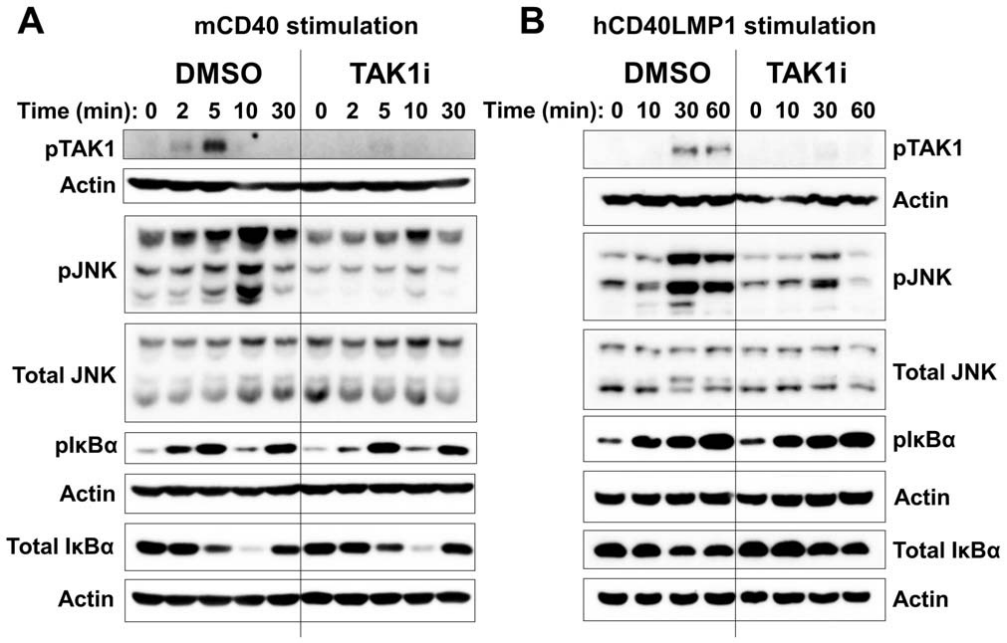
Role of TAK1 in CD40 and LMP1 early signaling events. A) Mouse CH12.LX B cells were treated with DMSO or TAK1i for 30 min prior to stimulation with anti-mCD40 Ab for 0, 2, 5, 10, or 30 min. Western blots show pTAK1, pJNK, pIκBα, and total IκBα, with total JNK and actin as loading controls. B) Similar to panel *A*, except that CH12.hCD40LMP1 mouse B cells were stimulated with anti-hCD40 Ab for 0, 10, 30, or 60 min. Data shown are representative of three independent experiments.

**Figure 4:**
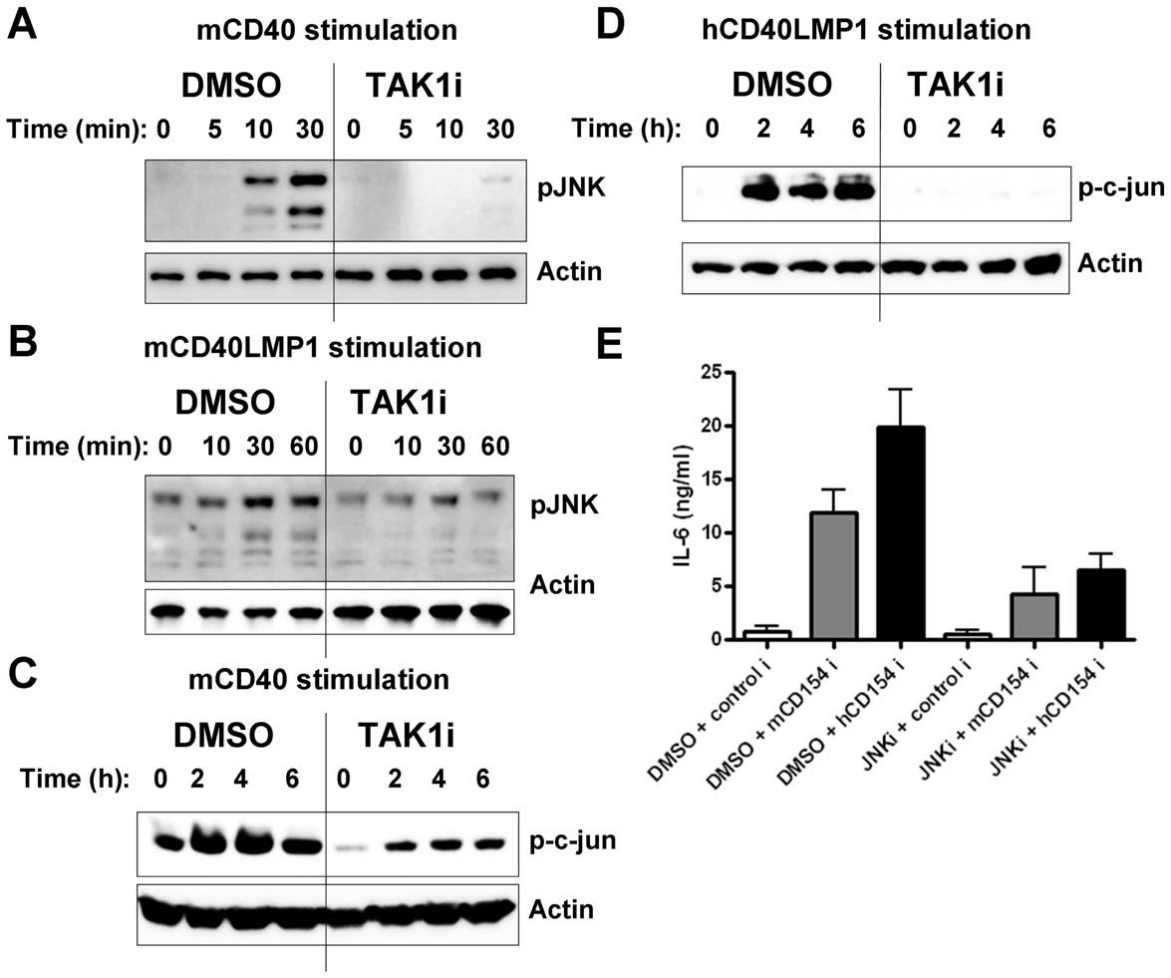
Requirement for TAK1 in CD40- and LMP1-mediated activation of the JNK pathway. A) WT mouse splenic B cells were treated with DMSO or TAK1i for 30 min prior to stimulation with anti-mCD40 Ab for 0, 5, 10, or 30 min. Western blots show protein levels for pJNK, with actin as a loading control. B) Similar to panel *A*, except that splenic B cells from a mCD40LMP1 Tg mouse were stimulated for 0, 10, 30, or 60 min. C) Mouse CH12.LX B cells were treated with DMSO or TAK1i for 30 min prior to stimulation with anti-mCD40 Ab for 0, 2, 4, or 6 h. Western blots show p-c-jun, with actin as a loading control. D) Similar to panel *C*, except that CH12.hCD40LMP1 mouse B cells were stimulated with anti-hCD40 Ab. Data shown are representative of at least two independent experiments. E) CH12.hCD40LMP1 cells were treated with DMSO or JNKi prior to stimulation with control insect cells (control i) or cells expressing either mCD154 (mCD154 i) or hCD154 (hCD154 i). Cells were cultured for 24 h and supernatants were collected for IL-6 ELISA assay. Data are mean values ± SEM of triplicate samples from two independent experiments.

Activation of both JNK and NF-κB pathways is required for CD40-induced IL-6 production in B cells [49], [50]. To examine the requirement for TAK1 in these early signaling pathways, mouse B cells were treated with DMSO or TAK1i prior to stimulation through either mCD40 or hCD40LMP1. Although TRAF6 is required for the activation of TAK1 and NF-κB by LMP1 [12], we found that TAK1 inhibition had no detectable effect on the two molecular events of IκBα phosphorylation and degradation (hallmarks of canonical NF-κB activation) induced by CD40 or LMP1 (Fig. 3). However, treatment with TAK1i greatly diminished both CD40- and LMP1-mediated JNK activation (Fig. 3). We observed similar results using primary mouse B cells (Fig. 4A & B).

JNK is a potent activator of the transcription factor c-jun, required for B cell IL-6 production by CD40 [49], [51], [52]. Treatment with TAK1i significantly reduced activation of c-jun by both CD40 and LMP1 (Fig. 4C & D), corroborating results for JNK activation. Additionally, LMP1 required JNK for IL-6 production (Fig. 4E), as previously shown for CD40 [50], [53].

### The role of TRAFs in TAK1 activation

**Figure 5:**
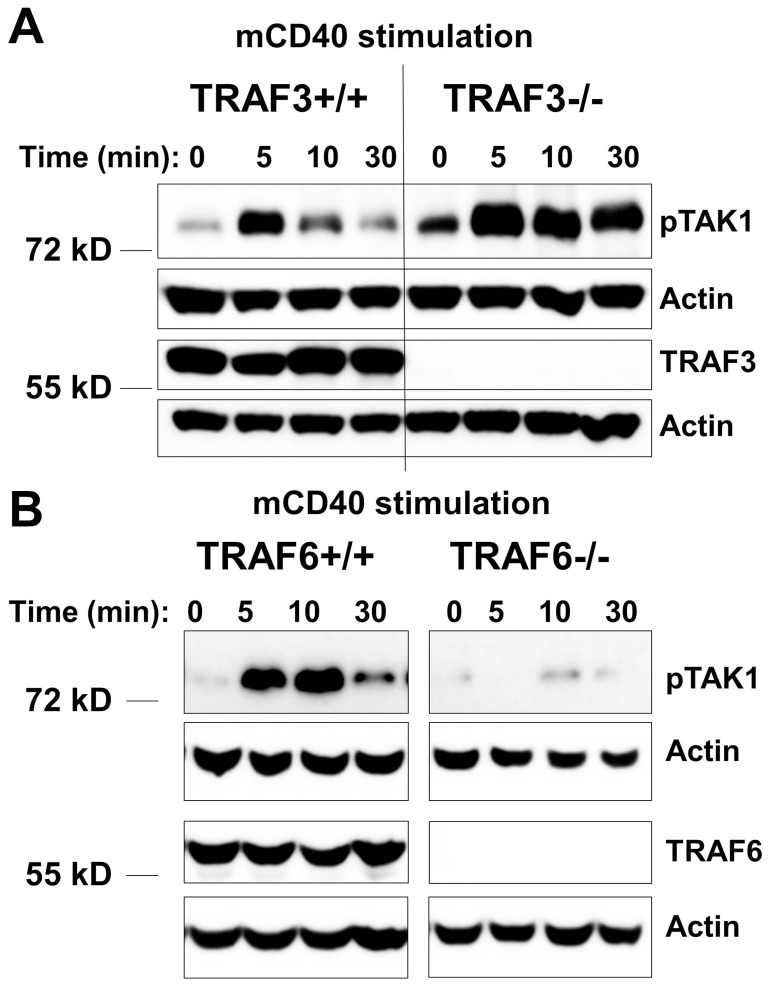
Requirement of TRAFs in CD40-mediated TAK1 activation. A) A20.TRAF3^+/+^ or A20.TRAF3^−/−^ mouse B cells were stimulated with anti-mCD40 Ab for 0, 5, 10, or 30 min. Western blots show pTAK1 and TRAF3, with actin as a loading control. B) Similar to panel *A*, except that A20.TRAF6^+/+^ or A20.TRAF6^−/−^ mouse B cells were used. Western blots show pTAK1 and TRAF6, with actin as a loading control. These images were part of the same membrane, but the middle lanes were omitted here for clarity. Data shown are representative of at least four independent experiments.

**Figure 6:**
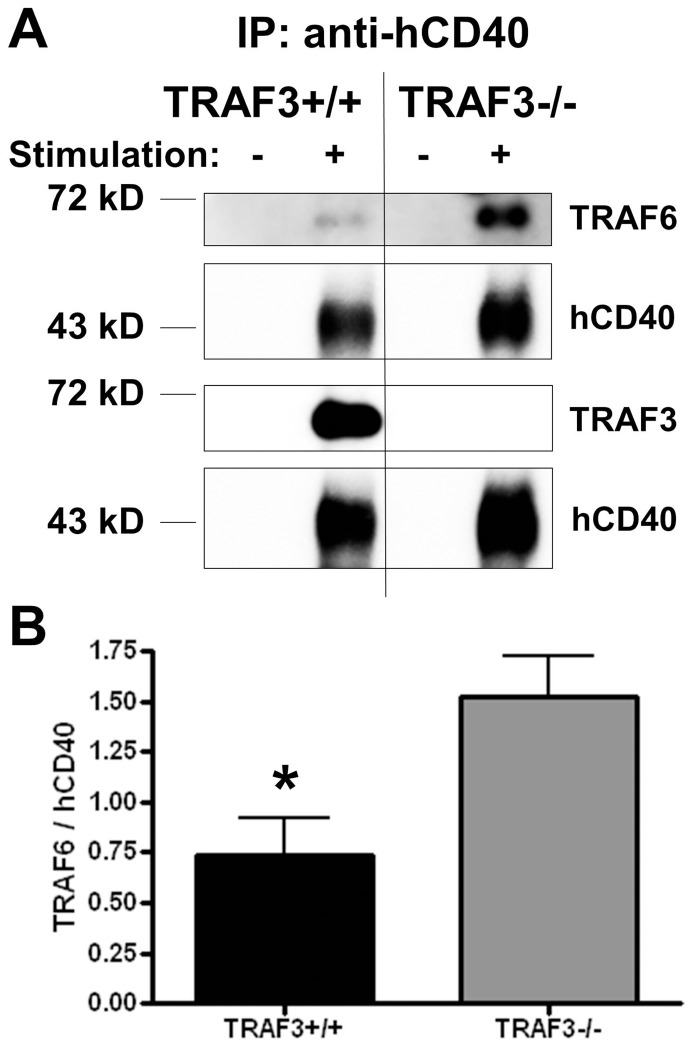
Association of TRAF6 with CD40 in the presence or absence of TRAF3. A) CH12.TRAF3^+/+^ or CH12.TRAF3^−/−^ mouse B cells expressing hCD40 were stimulated with control (−) or anti-hCD40 (+) Ab-coated beads for 10 min. hCD40 was immunoprecipitated using the same beads. B) Quantitation by luminescence imaging of the results of 3 independent experiments, mean values ± SEM. *p<0.05.

TRAF3, TRAF5, and TRAF6 positively regulate JNK and NF-κB activation by LMP1 in B cells [12], [18], [22], and we previously demonstrated a requirement for TRAF6 in LMP1-mediated TAK1 activation [12]. TRAF3 does not alter TRAF6 recruitment to LMP1 [12]. We also previously showed that TRAF5, which requires TRAF3 for LMP1 binding, plays no demonstrable role in LMP1-mediated TAK1 activation [12], [22]. TRAF6 plays a critical role in CD40-mediated JNK activation [29], and in contrast to its role in LMP1 signaling, TRAF3 negatively regulates JNK activation induced by CD40 [18]. However, the role of TRAFs in CD40-mediated TAK1 activation, as well as the effects of TRAFs on TRAF6 binding to CD40, were unknown. We thus examined the potential role of TRAFs 3 and 6 in CD40-mediated TAK1 activation, using TRAF3^−/−^ or TRAF6^−/−^ mouse B cell lines [18], [26], [29]. Subclones used all had similar levels of hCD40 expression as determined by flow cytometry (data not shown). The absence of TRAF3 was associated with enhanced TAK1 activation following CD40 stimulation, but in contrast TRAF6 was required (Fig. 5). It is important to note previous results demonstrating that TRAF3 deficiency has no effect on TRAF6 expression in B cells [18], a finding that was confirmed in the subclones used for these studies (data not shown). Interestingly, we found that the recruitment of TRAF6 to CD40 following CD40 engagement was enhanced in B cells deficient in TRAF3 (Fig. 6).

## Discussion

TRAF6 plays a critical role in signaling through both CD40 and LMP1, despite distinct mechanisms of association with the two molecules [12]. In the present study, we show that TAK1 was required for IL-6 production and JNK activation, but not for molecular events required for canonical NF-κB activation, by both CD40 and LMP1. Inasmuch as TRAF6 is required for the same events involved in LMP1-mediated NF-κB activation [12], this indicates that TRAF6 does not require TAK1 for all the LMP1-induced signals that it mediates. We also observed that TRAF3 inhibited both TRAF6 recruitment to, and TAK1 activation by CD40, but not LMP1, demonstrating that interactions between different TRAFs play distinct regulatory roles in CD40 vs. LMP1 signaling pathways. This raises the possibility of disrupting pathogenic LMP1 signaling while leaving normal CD40 signaling intact.

This study used complementary approaches of a TAK1 inhibitor and a TAK1 DN mutant to explore the requirement for TAK1 activation in TRAF6-dependent signaling and function. We also considered utilizing B cell-specific TAK1-deficient mice [40] bred to mCD40LMP1 transgenic mice [23]. However, upon obtaining these mice, we discovered that TAK1 expression in their B cells was ≥50% of normal levels (data not shown). Mice lacking TAK1 in B cells have a substantial reduction in total B cell numbers [54], suggesting that TAK1 plays an important role in normal B cell maturation, and thus B cells with inefficient TAK1 deletion may be favored to complete development. This complicates the interpretation of results using this *in vivo* model, and prevented us from employing these mice to address our hypothesis.

We demonstrated that TAK1 was required for IL-6 production following CD40 and LMP1 stimulation. Previous work showed that TRAF3 is not required for IL-6 production mediated by CD40 or LMP1 [18]. While TRAF5 contributes to LMP1-mediated IL-6 production [22], interestingly, the presence of the CY TRAF1/2/3/5/6 binding site is not required for this LMP1 function [25]. A CD40 mutant that does not bind TRAF6 cannot induce B cell IL-6 production [44]. TRAF6 associates with the TRAF1/2/3/5 binding site of LMP1, in contrast to its distinct binding site on CD40 [12], so disrupting only TRAF6 binding to LMP1 by mutating this site is not possible. However, given the critical role of TRAF6 in LMP1-mediated JNK and NF-κB activation [12], TRAF6 is likely required for IL-6 production by LMP1.

Although IL-6 is critical for many cellular functions, including B cell antibody production, dysregulation of IL-6 production has been implicated in the pathogenesis of numerous autoimmune/inflammatory diseases and cancers, including RA, SLE, inflammatory bowel disease, Crohn's disease, Castleman's disease, multiple myeloma, Hodgkin's disease, colon cancer, and cancer cachexia [55]–[57]. LMP1 is believed to play an important role in EBV-associated development and/or exacerbation of some of these pathologies [12]–[14]. Our results suggest TAK1 as a possible drug target in these diseases. Although a TAK1 inhibitor has not yet been approved for use in clinical trials, efforts are being made to modify/develop such inhibitors, as inhibition of TAK1 in preclinical models of colon and pancreatic cancer reduces tumor burden [35], [36].

A question raised by our findings is the precise mechanism by which TAK1 activates JNK following CD40 or LMP1 signals. JNK is directly phosphorylated by MKK4 and MKK7 [32], [46], [52], which are both phosphorylated by TAK1 [31], [34], [54], [58]. It is unclear whether either or both of these two MKKs is used by TAK1 in CD40 or LMP1 signaling, and whether CD40 and LMP1 require the same MKKs. Although TAK1i had no detectable effect on IκBα phosphorylation and degradation (events indicative of canonical NF-κB activation) by CD40 and LMP1, there are numerous additional MAP3Ks that participate in these NF-κB pathway events, including MEKK1, MEKK2, MEKK3, ASK1, Tpl-2, and GCK [19], [46]. CD40 and LMP1 may use the same or distinct kinase(s) to activate events in NF-κB pathways in a TAK1-independent manner.

TRAF6 is a key regulator of TAK1 activation by a variety of receptors, and associates with TAK1 [47], [59]. Findings presented here show for the first time a negative regulatory role for TRAF3 in TAK1 activation, as the presence of TRAF3 reduced the amount of TRAF6 at the receptor complex. TRAF3 may block TRAF6 recruitment to CD40 directly by competing with TRAF6 for binding, or via steric hindrance, although they do not share a direct binding site. CD40 has three known TRAF binding sites in its CY domain: QEPQEINF (TRAF6), PVQET (TRAF1/2/3/5), and a potential second TRAF2 binding site (SVQERQ) [12], [24], [60]. The TRAF6 and TRAF1/2/3/5 binding sites are in close proximity. Point mutations in the TRAF6 binding site affect TRAF1/2/3/5 binding and TRAFs 1, 2, and 3 make contacts in the TRAF6 binding site region [61]. Together, these data support the possibility that TRAF3 can block TRAF6 binding to CD40. Activation of CD40 results in the rapid recruitment of TRAF2 and TRAF3 to the plasma membrane, where they associate with CD40 in lipid rafts [62]. Little TRAF6 is recruited compared to TRAF3 [62], consistent with the idea that TRAF3 inhibits recruitment of TRAF6. Another possibility is that TRAF3 sequesters TRAF6 in the cytoplasm, limiting the amount of TRAF6 available for binding to CD40, as TRAF molecules form homo- and hetero-oligomers [63], [64]. Finally, TRAF3 could recruit other non-TRAF proteins that interfere with or prevent TRAF6 recruitment to CD40.

Understanding the specific nature of how CD40 and LMP1 use TAK1 may be useful for designing therapies that target the pathogenic effects of LMP1. LMP1 itself may be a poor therapeutic target, as it lacks a traditional EC domain and is constantly processed from the cell surface [16]–[18], [24]. Thus, there is interest in disrupting downstream LMP1 signaling. Elucidating the role of TAK1 in CD40 and LMP1 functions increases our understanding of how LMP1 drives B cell pathogenesis. Here, we have shown that TAK1 played a critical role in JNK-mediated IL-6 production, so it may be possible to disrupt LMP1 signaling by targeting TAK1. TAK1 inhibition has shown substantial promise in killing colon and pancreatic cancer cells [35], [36], and data here suggest that TAK1 inhibition, possibly in combination with other therapies, could be effective in preventing LMP1-mediated B cell pathogenesis.

## Materials and Methods

### Mouse splenic B cells

C57BL/6 mice were obtained from Jackson Laboratories (Bar Harbor, ME). The mCD40LMP1 Tg mice were described previously [23]. Briefly, expression of the mCD40LMP1 transgenic receptor is driven by the MHC II promoter, as CD40 and MHC II expression in immune cells largely coincides [23]. The mCD40LMP1 Tg mice were bred to CD40^−/−^ mice so that endogenous ligand for CD40, CD154, induces only mCD40LMP1 signaling [23]. Splenic B cells were first isolated by density gradient centrifugation through a 55%:65%:75% Percoll gradient. Lymphocytes at the interphase between 55% and 65% and between 65% and 75% were collected, and B cells were further purified by negative selection using MACS mouse CD43 (Ly-48) MicroBeads, MACS LD separation columns, and MidiMACS separator (Miltenyi Biotec, Auburn, CA) according to the manufacturer's protocols. The purity of isolated B cells was monitored by FACS analysis using FITC-anti-mouse B220 Ab and FITC-anti-rat IgG Ab (eBioscience, San Diego, CA). B cell purity was ≥99%. Flow cytometry was performed on a Guava EasyCyte using CytoSoft software (Guava Technologies Inc, Hayward, CA). Data were analyzed using FlowJo software (Tree Star, Ashland, OR). All mice were housed in a pathogen-free barrier facility with restricted access, and all procedures were performed as approved by the University of Iowa Animal Care and Use Committee, Iowa City, IA, which approved this study.

### Human B cells

Peripheral blood mononuclear cells were isolated from a leukocyte reduction system (LRS) cone (DeGowin Blood Center, University of Iowa Hospitals and Clinics, Iowa City, IA) as previously described [65], using lymphocyte separation medium (Mediatech Inc, Manassas, VA). B cells were purified using the StemSep negative selection human B cell enrichment kit (STEMCELL Technologies, Vancouver, BC, Canada) and the MidiMACS separator and MACS LS separation columns (Miltenyi Biotec) per the manufacturers' protocols. The purity of isolated B cells was determined by FACS analysis as described above using PerCP-anti-human CD19 and PerCP-anti-rat IgG Abs (BioLegend and BD Pharmingen, San Diego, CA). B cell purity was >90%. The use of de-identified samples from healthy adult volunteers was approved by the University of Iowa Institutional Review Board, which approved this study.

### Cell lines

The mouse B cell lines A20.2J and CH12.LX have been described previously [66], [67]. The following TRAF-deficient and/or stably transfected subclones were used: A20.TRAF6^−/−^ [29], A20.TRAF3^−/−^ [18], CH12.TRAF3^−/−^ [26], A20.hCD40LMP1 and CH12.hCD40LMP1 [16], [20], [45], CH12.hCD40 [20], [68], CH12.hCD40.DN-TAK1 and CH12.hCD40LMP1.DN-TAK1 [2], [29], [30], [46], [47]. These cell lines were maintained in RPMI 1640 medium (Invitrogen, Grand Island, NY) with 10 uM 2-mercaptoethanol (Invitrogen), 10% heat-inactivated fetal calf serum (FCS; Atlanta Biologicals, Atlanta, GA), and antibiotics (Invitrogen) (B cell medium; BCM10). The cell lines expressing hCD40LMP1 were maintained in 400 ug/ml G418 disulfate (Research Products International, Mt. Prospect, IL). The cell lines expressing DN-TAK1 were maintained in 400 ug/ml G418 disulfate, 200 ug/ml hygromycin (Invitrogen), and 400 ug/ml zeocin (Research Products International).

### Reagents and antibodies

Percoll density gradient solution was purchased from GE Healthcare (Uppsala, Sweden). Dimethyl sulfoxide (DMSO) was purchased from Fisher Scientific (Rockford, IL). 5Z-7-Oxozeaenol (TAK1 kinase inhibitor) was purchased from Sigma-Aldrich (St. Louis, MO). JNK inhibitor VIII (JNKi) was purchased from Calbiochem (Rockland, MA). PageRuler prestained protein ladder electrophoresis size marker was purchased from Fermentas Inc (Glen Burnie, MD). Disuccinimidyl suberate (DSS) was purchased from Thermo Scientific (Rockford, IL). Isopropyl-β-D-thiogalactopyranoside (IPTG) was purchased from Amresco (Solon, OH). The following antibodies were used for cell stimulation: HM40.3 (hamster anti-mouse CD40 Ab; eBioscience), G28.5 (produced from a hybridoma producing mouse IgG1 anti-hCD40 mAb; American Type Culture Collection, Manassas, VA), 1C10 (produced from a hybridoma producing rat IgG2a anti-mCD40 mAb; a gift from Dr. Frances Lund, University of Rochester, Rochester, NY) and MOPC-21 Ab (mouse IgG1, BioLegend, San Diego, CA). The following antibodies were used for ELISAs: rat anti-mouse IL-6 Ab, rat anti-human IL-6 Ab, biotin rat anti-mouse IL-6 Ab, and biotin rat anti-human IL-6 Ab (eBioscience). Recombinant mouse and human IL-6 were purchased from PeproTech Inc (Rocky Hill, NJ). The following primary antibodies were used for Western blotting: rabbit anti-phospho-JNK Ab, rabbit anti-phospho-TAK1 Ab, rabbit anti-phospho-IκBα Ab, rabbit anti-total IκBα Ab, and rabbit anti-phospho-c-jun Ab (Cell Signaling Technology, Danvers, MA), rabbit anti-total JNK Ab and rabbit anti-TRAF3 Ab (Santa Cruz Biotechnology, Santa Cruz, CA), mouse anti-actin Ab (Millipore, Billerica, MA or Sigma-Aldrich), rabbit anti-TRAF2 Ab, chicken anti-TRAF6 Ab, and hamster anti-TRAF6 Ab (Medical and Biological Laboratories, Japan), and S12 (mouse anti-LMP1 IgG mAb; produced from a hybridoma that was a gift from Dr. Fred Wang, Harvard University, Cambridge, MA). The sheep anti-hCD40 Ab was generated in our laboratory. The secondary Abs used included goat anti-mouse IgG, goat anti-rabbit IgG, goat anti-chicken IgG, and donkey anti-sheep IgG (Jackson ImmunoResearch Laboratories), and goat anti-hamster IgG (Santa Cruz Biotechnology). Hi5 insect cells infected with either wildtype (WT) or recombinant baculovirus encoding mouse or human CD154 were previously described [69], [70].

### Enzyme-linked immunosorbent assays (ELISAs)

Cells (2×10^5^ mouse B cells, 1×10^5^ human B cells or mouse B cell lines) were resuspended in 100 ul BCM10 in a 96-well tissue culture plate. The cells were treated with DMSO or either 5Z-7-Oxozeaenol (100 nM) or JNK inhibitor VIII (10 uM) (concentration of DMSO in samples = 1%), followed by stimulation with Hi5 insect cells expressing WT baculovirus (control), mCD154, or hCD154 at a 1∶5 insect-cell∶B-cell ratio for 24 h at 37°C. CH12.DN-TAK1 cells were incubated in the presence or absence of IPTG (100 uM) overnight prior to the start of the experiment, and cells that were treated with IPTG were cultured in the presence of 100 uM IPTG for the duration of the assay. Supernatant was collected, and an IL-6 ELISA was performed as previously described [23], [48].

### Antibody production

CH12.LX cells produce IgM reactive with phosphatidylcholine, present on erythrocyte membranes, and IgM-producing CH12.LX cells were enumerated by hemolytic plaque assay as previously described [18], [21], [71]. Ab-producing cells were measured as cells that formed lytic plaques on a lawn of SRBCs (Elmira Biologicals, Iowa City, IA) and were quantified as plaque-forming cells (Pfcs) per 10^6^ viable cells. CH12.hCD40 or CH12.hCD40LMP1 cells were stimulated at 1.5×10^3^/well in 96-well plates in 200 ul medium for 72 h. DN-TAK1 expression was induced by preincubation with IPTG for 18 h prior to addition of stimuli.

### Signaling assays

1–2×10^6^ cells were washed in RPMI 1640 medium, resuspended in 1 ml BCM10 in 1.5 ml Eppendorf tubes, and rested for 45 min at 37°C. Cells were then stimulated for the indicated time periods with anti-hCD40 Ab (G28.5, 10 ug/ml) or anti-mCD40 Ab (1C10 or HM40.3, 10 ug/ml). For studies involving TAK1 inhibition, cells were treated with DMSO or 5Z-7-Oxozeaenol (100 nM) (concentration of DMSO in samples = 1%) for 30 min prior to receptor stimulation, with the inhibitor present for the duration of the assay. Whole cell lysates were prepared as previously described [12].

### Western blots

10–15 ul of sample were resolved on 10% SDS-PAGE. Proteins were transferred to Immobilon-P PVDF membranes (Millipore). Membranes were blocked with 10% dry milk in Tris-buffered saline with Tween 20 (TBST; 3% 5 M NaCl, 1% 1 M Tris, and 0.1% Tween 20 in H_2_O) for 1 h, washed in TBST, incubated with primary Abs overnight, washed in 10% dry milk in TBST, and incubated with secondary Abs for 1–2 h or overnight and developed as previously described [12].

### Activation of c-jun

3×10^6^ cells were resuspended in 3 ml of BCM10, added to a 6-well tissue culture plate, and rested for 45 min at 37°C. The cells were treated with DMSO or 5Z-7-Oxoaeaenol (100 nM) for 30 min, followed by stimulation with 10 ug/ml G28.5 or HM40.3 mAbs, then incubated for 2, 4, or 6 h at 37°C. After chilling plates to 4°C, cells and medium were transferred into 1.5 ml Eppendorf tubes and whole cell lysates were prepared as previously described [12].

### Immunoprecipitation

Dynal protein G magnetic beads (Invitrogen) were coated with G28.5 or MOPC-21 Ab (10 ug/10 ul beads) as described previously [29]. Abs were conjugated to the beads using DSS according to the manufacturer's protocols. 2×10^7^ cells were incubated in 1 ml of BCM10 with Ab-coated beads for 10 min at 37°C. Beads and cells were then pelleted and lysed as described previously [29]. Bead-bound proteins were resuspended in 2× SDS-PAGE loading buffer and boiled for 10 min at 95°C.

### Statistical analysis

Statistical comparisons were done using a two-sided unpaired Student's *t* test. P-values<0.05 were considered statistically significant.
